# Silver nanoparticles induced hepatoxicity via the apoptotic/antiapoptotic pathway with activation of *TGFβ-1* and *α-SMA* triggered liver fibrosis in Sprague Dawley rats

**DOI:** 10.1007/s11356-022-21388-3

**Published:** 2022-06-18

**Authors:** Doaa H. Assar, Abd-Allah A. Mokhbatly, Emad W. Ghazy, Zizy I. Elbialy, Ahmed A. Gaber, Ayman A. Hassan, Ahmed Nabil, Samah Abou Asa

**Affiliations:** 1 Clinical Pathology Department, Faculty of Veterinary Medicine, Kafrelsheikh University, Kafrelsheikh, 33516 Egypt; 2 Department of Fish Processing and Biotechnology, Faculty of Aquatic and Fisheries Sciences, Kafrelsheikh University, Kafrelsheikh, 33516 Egypt; 3 High Technological Institute of Applied Health Sciences, Egypt Liver Research Institute and Hospital (ELRIAH), Sherbin, El Mansora Egypt; 4 Beni-Suef University, Beni-Suef, Egypt; 5 Egypt Liver Research Institute and Hospital (ELRIAH), Sherbin, El Mansora Egypt; 6 Pathology Department, Faculty of Veterinary Medicine, Kafrelsheikh University, Kafrelsheikh, 33516 Egypt

**Keywords:** Silver nanoparticles, Hepatotoxicity, Fibrosis, Apoptotic pathway, *TGFβ*, *α-SMA*

## Abstract

Despite the extraordinary use of silver nanoparticles (AgNPs) in medicinal purposes and the food industry, there is rising worry about potential hazards to human health and the environment. The existing study aims to assess the hepatotoxic effects of different dosages of AgNPs by evaluating hematobiochemical parameters, oxidative stress, liver morphological alterations, immunohistochemical staining, and gene expression to clarify the mechanism of AgNPs’ hepatic toxic potential. Forty male Sprague Dawley rats were randomly assigned into control and three AgNPs intraperitoneally treated groups 0.25, 0.5, and 1 mg/kg b.w. daily for 15 and 30 days. AgNP exposure reduced body weight, caused haematological abnormalities, and enhanced hepatic oxidative and nitrosative stress with depletion of the hepatic GSH level. Serum hepatic injury biomarkers with pathological hepatic lesions where cholangiopathy emerges as the main hepatic alteration in a dosage- and duration-dependent manner were also elevated. Furthermore, immunohistochemical labelling of apoptotic markers demonstrated that Bcl-2 was significantly downregulated while caspase-3 was significantly upregulated. In conclusion, the hepatotoxic impact of AgNPs may be regulated by two mechanisms, implying the apoptotic/antiapoptotic pathway via raising BAX and inhibiting Bcl-2 expression levels in a dose-dependent manner. The TGF-β1 and α-SMA pathway which triggered fibrosis with incorporation of iNOS which consequently activates the inflammatory process were also elevated. To our knowledge, there has been no prior report on the experimental administration of AgNPs in three different dosages for short and long durations in rats with the assessment of Bcl-2, BAX, iNOS, TGF-β1, and α-SMA gene expressions.

**Graphical abstract:**

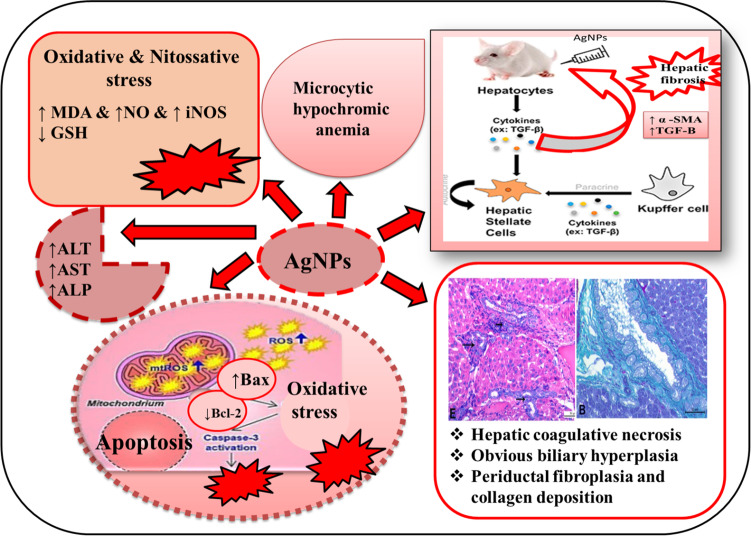

## Introduction

Nanotechnology is widely used in various applications of nutrition, therapy and medication with incorporation into consumer products, for example, food packaging material, food supplements, textiles, and spray products, because of their antimicrobial properties as well as potent anti-inflammatory, antiviral and/or anticancer activities (Wei et al. 2015; Lee and Jun 2019; Paladini and Pollini 2019), making it likely that the biomedical applications of these nanoparticles (NPs) will expand in the future. Hence, increased consumer exposure to silver nanoparticle (AgNP)–containing products has raised the potential need for their toxicological assessment (Rosario et al. 2020). Several in vitro studies have shown that AgNPs can induce cytotoxicity, DNA damage, oxidative stress (OS) and inflammatory responses in human cells (Ahamed et al. 2008; Ahamed et al. 2010; Rosario et al. 2016; Carrola et al. 2016; Bastos et al. 2016; Akter et al. 2017; Rosario et al. 2018), but it still has not been clearly identified yet, and few studies have studied on the toxic effects of sublethal doses exposed to AgNPs for a short period (Lee et al. 2012; Lamberti et al. 2014). In earlier studies, Takenaka et al. (2001) and Arora et al. (2009) reported that the liver appears to be a major accumulation site of circulatory AgNPs, as a significant quantity of AgNPs is detected in the liver of rats following a 90-day oral administration (Kim et al. 2010). A recent clinical report also described the absorption of AgNPs into the circulation following the use of AgNP-coated dressings for burns (Vlachou et al. 2007; Ferdous and Nemmar 2020). Van der Zande et al. (2012) recorded that the liver is from the major organs of AgNPs (De Jong et al. 2013). The excessive accumulation of AgNPs in the liver led to severe pathological changes (Lee et al. 2013a, b). Also, AgNPs have an important role in reactive oxygen species (ROS) induction in many body cells (Choi et al. 2009). ROS are continually produced and removed in biological systems by endogenous or exogenous antioxidants (Mohammadi et al. 2013; Piao et al. 2011), but excessive generation of ROS can lead to apoptosis and cause oxidative DNA damage (Xu et al. 2012). The purpose of the existing study was to assess the impact of different doses of AgNPs on body performance, haemato-biochemical parameters, oxidative stress/antioxidant status, hepatic morphological alterations, apoptotic/antiapoptotic pathway and the underlying molecular mechanisms after 15 and 30 days of intraperitoneal injection of AgNPs.

## Materials and methods

### Ethical statement

The experiment was approved by the Institutional Animal Care and Animal Ethics Committee, Faculty of Veterinary Medicine, Kafrelsheikh University, Egypt. All precautions were followed to diminish animal suffering during the experiment (KFS2020-3).

### Silver nanoparticle synthesis

Silver nanoparticles were synthesized as follows: 25 mL of 6.8 mM trisodium citrate in an aqueous solution, containing 7 μM of tannic acid, was heated to 60 °C in an oil bath. After heating, the solution was added directly with strong stirring to 100 mL of 0.74 mM AgNO_3_ which was also pre-heated to 60 °C. This mixture was kept at 60 °C for a few minutes until the colour of the solution turned to yellow. The mixture was then kept at 97 °C for a further 45 min followed by cooling down to room temperature and finally stored in the dark at 4 °C (Bastús et al. 2014).

### Characterization of silver nanoparticles

Characterization of AgNPs is important in order to evaluate the functional aspects of the synthesized particles. We characterize our prepared AgNPs by two different analytical techniques: dynamic light scattering (DLS) and transmission electron microscopy (TEM).

#### Dynamic light scattering

It is a method that depends on the interaction of light with particles. It can be used for the measurement of narrow particle size distributions, especially in the range of 2–500 nm. It is mainly used to determine particle size and size distributions in aqueous or physiological solutions. The size obtained from DLS is usually larger than TEM, which may be due to the influence of Brownian motion (Zhang et al. 2016). We use the Malvern Zetasizer Nano series at the Electron Microscopy Unit, Mansoura University, Egypt.

#### Transmission electron microscopy

Samples were loaded on carbon-coated Cu grids (200 mesh) and examined by JEM 2100 electron microscope (JEOL, Tokyo) at 200 kV using HRTEM and ORIUS Gating camera at the Electron Microscopy Unit, Mansoura University, Egypt.

### Experimental animals

Forty male Sprague Dawley rats (10 weeks old weighing 150 ± 20 g) were provided by the Medical Experimental Research Center (MERC) of Mansoura University. They were housed in stainless-steel cages containing sterile paddy husk as bedding material in ventilated animal rooms. They were acclimated for 1 week, prior to starting the experiment, in a controlled environment (temperature 24 ± 2 °C; humidity 60 ± 10% and light 12 h light–dark cycle) with free access to water and a standard pellet diet ad libitum. The experimental protocol was approved by Kafrelsheikh University Faculty of Veterinary Medicine according to the guidelines approved by the Institutional Animal Ethical Clearance (IAEC) committee.

### Experimental protocol

The experimental animals after a 1-week adaptation period were equally divided into 4 groups (10 rats per group) as follows:

1. Control group: received 0.9% normal saline via i.p administration
2. Low–AgNP dose group (0.25 mg AgNPs /kg b.w.).
3. Medium–AgNP dose group (0.5 mg AgNPs /kg b.w.)
4. High–AgNP dose group (1.0 mg AgNPs /kg b.w.)

Each experimental group was subdivided in into 2 subgroups; each subgroup consists of 5 rats. The 1st subgroup was injected with an AgNP dose daily for 15 successive days, and the 2nd subgroup was injected with an AgNP dose for 30 successive days.

**Fig. 1.**
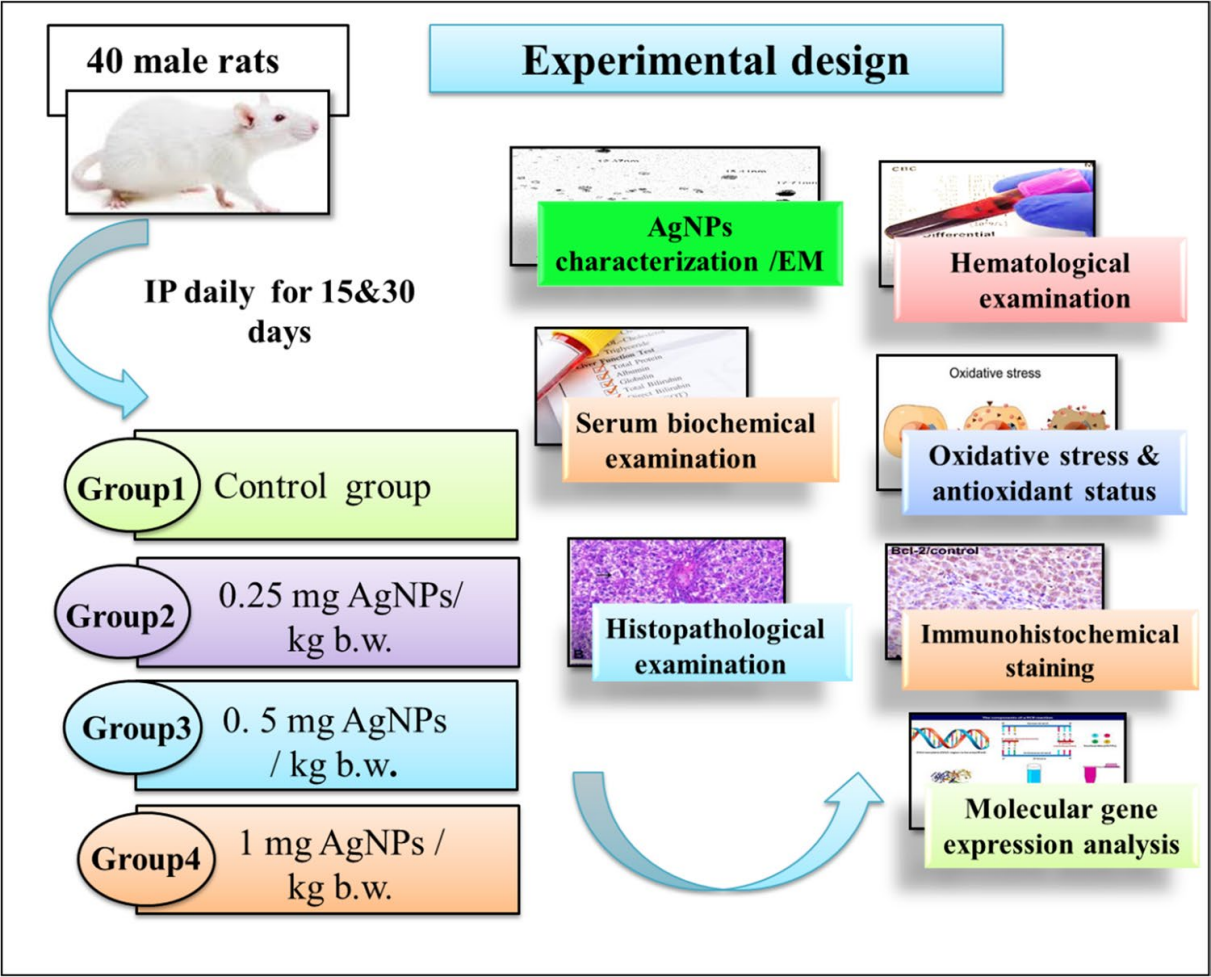
Experimental design

N.B: The animals were exposed to AgNPs (suspended in 0.9% normal saline) via i.p administration. The NPs were freshly prepared every day based on rat b.w. and immediately used. The levels of used doses were selected according to Qin et al. (2017). The experimental scheme is shown in Fig. 1.

### Sampling

At the end of 15 and 30 days post treatment, animals were weighed then euthanatized with 75 mg/kg b.w thiopental sodium. Two separate blood samples were collected from each rat; the first blood sample was taken with anticoagulant (K_2_EDTA) for haematological analysis. The second sample was gathered in plain tubes, lifted to clot, then centrifuged at 3000 rpm for 10 min for serum separation and stored in Eppendorf tubes at −80 °C until used for serum biochemical analysis (Coles 1986). The livers were dissected out and weighted. Two hepatic tissue specimens were taken from each rat. The 1st tissue specimen was kept in formalin 10% for histopathological and immunohistochemical examination. The 2nd specimen was preserved at −80 °C for evaluating oxidative stress, antioxidant status and molecular gene expression analysis.

### Haematological examination

Anti-coagulated blood samples were analysed for measuring the RBC number, Hb and PCV, RBC indices including mean corpuscular volume (MCV), mean corpuscular haemoglobin (MCH), mean corpuscular haemoglobin concentration (MCHC), platelet count (PLTs), WBC and differential leucocyte count for neutrophils, lymphocytes, monocytes, eosinophils and basophils using a blood cell counter (CELL-DYN Ruby, Abbott, USA) with its kits (Diluent/Sheath, WBC Lyse and HGB lyse).

### Serum biochemical analysis

Serum alanine aminotransferase (ALT) and aspartate aminotransferase (AST) were estimated according to Bergmeyer et al. (1986), and alkaline phosphatase (ALP) was determined according to Schumann et al. (2011) using commercial kits Bio Diagnostic Co. (Giza, Egypt) using an autoanalyzer (Cobas INTEGRA 400 plus analyser).

### Hepatic oxidative stress and antioxidant biomarker estimation

The frozen liver tissues (about 1 g) were thawed then washed with ice-cold KCl solution (1.15%), blotted, weighed and then homogenized separately in 4 volumes of ice-cold homogenizing buffer (1.15% KCl and 50 nM Tris–HCl to adjust the pH at 7.4). About 1 g tissue from each sample was homogenized separately and centrifuged at 10,000g for 20 min (Sigma 2-16K). The supernatant was then separated, decanted and stored at −20 °C. Lipid peroxide was measured after the reaction with thiobarbituric acid and expressed as nanomole (nmol) MDA (malondialdehyde) per tissue weight according to Satoh (1978). NO (nitric oxide) was determined in hepatic tissue homogenate according to the method of Montgomery and Dymock (1961), while GSH (reduced glutathione) was also evaluated according to the method of Beutler et al. (1963).

### Histopathological examination

Tissue sections from the liver sampled from each rat were taken and processed for histopathological examination. The sections were immediately fixed in 10% formalin solution, dehydrated in alcohols, then cleared in xylene, and embedded in paraffin blocks. Sections of 5-μm thickness were prepared and stained with haematoxylin and eosin (Bancroft and Layton 2013) and Masson’s trichrome stain (Dries 2008).

### Immunohistochemical analysis of Bcl-2 and caspase-3 in hepatic tissues

Immunohistochemical staining of Bcl-2 and caspase-3 was performed on the hepatic tissue samples of rats that received AgNPs after 30 days of exposure using 4-μm-thick paraffin-embedded sections. The sections were deparaffinized in xylene and rehydrated in graded ethanol. For antigen retrieval, the sections were immersed in a solution of 0.05 M citrate buffer, pH 6.8. Endogenous peroxidase was blocked by incubation in 0.3% H_2_O_2_ in methanol for 20 min at room temperature (RT). To prevent binding of nonspecific proteins, the sections were treated with Protein Block Serum Free for 30 min at RT. Immunolabelling was performed using rabbit monoclonal anti-BCL2 (Abcam, Cat# ab182858, at a dilution of 1: 500) and polyclonal anti-caspase 3 antibodies (Invitrogen, Cat# PA5-77887, at a dilution of 1:100) overnight in a humidified chamber at 4 °C. The sections were washed with phosphate-buffered saline (PBS) and were incubated with a goat anti-rabbit secondary antibody (Cat# K4003, EnVision+™ System Horseradish Peroxidase Labelled Polymer; Dako) for 30 min at room temperature. After washing 3 times with PBS, 3,3-diaminobenzidine tetrahydrochloride (Liquid DAB + Substrate Chromogen System, DakoR) was added to the sections. The sections were then washed in distilled water, counterstained with Mayer’s haematoxylin, dehydrated in an alcohol gradient, cleared with xylene and mounted for examination under light microscope (Saber et al. 2019). By counting 1000 cells in 10 high-power fields (×400), the ratio of hepatocytes with positive Bcl-2 and caspase-3 labelling was calculated. The immunoreactivities were scored as follows: negative, 0–10% positive cells; weakly positive, 10–25% positive cells; moderately positive, 25–50% positive cells; and strongly positive, >50% positive cells according to Abou Asa (2017). The proportion of positive cases of Bcl-2 and Caspase-3 was compared using the *T*-test, and *P* < 0.05 was considered significant.

### RNA extraction and qRT-PCR

Approximately 100 mg of hepatic tissues from all collected samples was rinsed in sterilized phosphate-buffered saline and homogenized in liquid nitrogen using a Teflon and pestle homogenizer. Total RNA was isolated using TRIzol (iNtRON Biotechnology) according to the manufacturer’s instructions. cDNA was synthesized from purified RNA using the Maxime RT PreMix (Oligo dT primer) (iNtRON Biotechnology, Korea). The reaction mixture including RNA and master mix was placed at 45 °C, then inactivated at 95 °C. qRT-PCR for the target genes was performed using the SensiFAST SYBR Lo-ROX Kit (Bioline) Master Mix. The primer sequences of the target and reference genes as well as the PCR conditions are mentioned in Table 1. The fold change of the mRNA expression level was calculated after recording the Ct values for both reference and target genes using the 2^−ΔΔCt^ method (Livak and Schmittgen 2001).

**Table 1:** Primer sequences used in qRT-PCR

Gene	Primer sequence 5′–3′	NCBI accession number	Reference
GAPDH*	F:CAGCAATGCATCCTGCAC R:GAGTTGCTGTTGAAGTCACAGG	XM_017592435.1	Nakahara et al. (2003)
TGFβ1	F-CCAGATCCTGTCCAAACTAA R-TTTTGTCATAGATTGCGTTG	X52498.1	Zhu et al. (2008)
iNOS	F-CTACCTACCTGGGGAACACCTGGG R-GGAGGAGCTGATGGAGTAGTAGCGG	S71597.1	Hori et al. (2001)
αSMA	F-CGATAGAACACGGCATCATC R-CATCAGGCAGTTCGTAGCTC	NM_031004.2	Ghassemifar et al. (1997)
BAX	F-GTTGCCCTCTTCTACTTTGC R-ATGGTCACTGTCTGCCATG	NM_017059.2	Sadek et al. (2018)
BCl-2	F-CCCCAGAAGAAACTGAACC R-GCATCTCCTTGTCTACGC	NM_016993.1	

GAPDH* as internal reference gene

### Statistical analysis

The obtained data were statistically analysed using SPSS version 22 using the *t* test; all values were represented as means (±) standard deviations (SD). The ANOVA test was used, and *P* ≤ 0.05 was considered statistically significant and *P* ≤ 0.001 highly significant according to Levesque (2007). The values were represented by the letters a, b, c and d, with the highest value being a.

## Results

### Characterization of the applied AgNPs

**Fig. 2.**
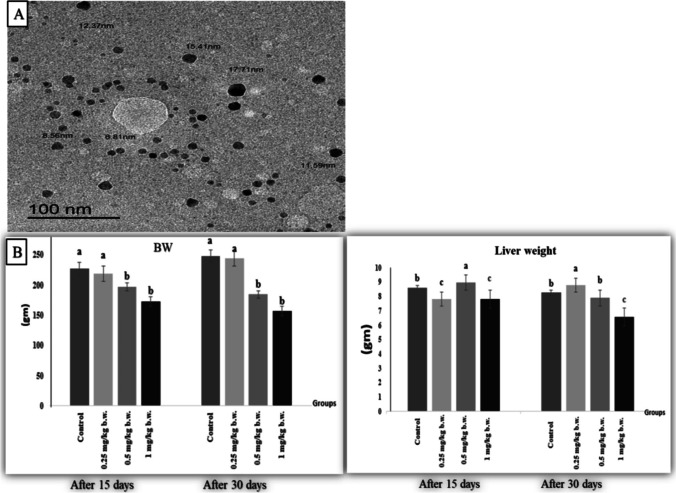
**a** Transmission electron micrograph showed AgNPs. The scale bar represents 100 nm. **b** Effect of AgNPs on body weight: BW and liver weight after 15 days and 30 days of i.p. injection

The form and size of AgNPs in deionized water were observed and photographed as shown in Fig. 2a. A laser diffraction particle size analyser was used to determine the distribution of AgNPs in the suspension (Zetasizer Nano ZS90, Malvern, UK). The scan showed AgNPs with a spherical and an average size of 12.0 nm.

### Body and liver weights

In terms of body weight changes, rats injected with 0.25 mg AgNPs /kg b.w. showed a non-significant change in the body weight throughout the trial when compared to the control rats. However, rats given 0.5 or 1 mg AgNPs had a substantial (*P* ≤ 0.05) drop in bw, which was more severe with greater dose and longer duration when compared to the control group (Fig. 2b). In terms of liver weight, rats that received 0.25 mg AGNPs had a significant decline (*P* < 0.05) in liver weight after 15 days followed by a significant increase (*P* < 0.05) after 30 days whereas rats given 0.5 mg AGNPs had the opposite effect when compared to the control group. Furthermore, rats injected with 1 mg AgNPs for the 15 and 30 days had a substantial reduction (*P* < 0.05) in liver weights (Fig. 2b).

### Haematological findings

The existence of anaemia was demonstrated by a substantial (*P* ≤ 0.05) decrease in RBC count, Hb and HCT in all AgNP-treated groups. When compared to the control group, RBC indices revealed microcytic hypochromic anaemia, which was more apparent in the rat group that received 1 mg AgNPs in a time-dependent manner. Furthermore, when compared to the control group, RBC indices in the rats injected with 0.25 and 0.5 mg AgNPs after 15 and 30 days were not statistically different. Moreover, as compared to the control group, the PLT number of the 0.25-mg and 0.5-mg AgNP-treated groups rose considerably (*P* ≤ 0.05) in a dose- and time-dependent manner. However, rats treated with 1 mg AgNPs/kg showed no significant differences in PLT count when compared to the control group. Table 2 shows the summarized erythrogram results.

**Table 2:** Hematological findings of control and treated groups after 15 and 30 days. Mean values ± SEM

Groups	Group 1 (control)		Group 2 (0.25 mg/kg b.w.)		Group 3 (0.5 mg/kg b.w.)		Group 4 (1.0 mg/kg b.w.)		*P* value
	After15 days	After30 days	After15 days	After 30 days	After 15 days	After 30 days	After 15 days	After 30 days	
RBCs (10^6^/μl)	7.36 ± 0.43 a	7.82 ± 0.95 a	6.65 ± 0.44 b	6.68 ± 0.26 b	6.30 ± 0.25 b	6.49 ± 0.53 b	4.45 ± 2.54 c	4.70 ± 2.70 c	0.01
Hb (gm/dl)	13.38 ± 0.43 a	14.16 ± 0.93 a	12.14 ± 1.03 b	12.52 ± 0.58 b	11.94 ± 0.38 b	12.98 ± 1.59 b	8.34 ± 4.75 c	8.36 ± 4.88 c	0.01
HCT (%)	42.42 ± 0.95 a	44.7 ± 3.43 a	39.1 ± 2.81 b	40.26 ± 1.19 b	38.22 ± 1.20 b	40.32 ± 3.22 b	26.66 ± 15.18 c	27.74 ± 16.14 c	0.01
MCV (fl)	58 ± 3.22 a	57.42 ± 2.62 a	58.78 ± 1.96 a	60.34 ± 0.76 a	60.62 ± 1.74 a	62.3 ± 5.43 a	47.88 ± 26.77 b	47.16 ± 26.53 b	0.05
MCH (pg)	18.28 ± 1.19 a	18.18 ± 0.98 a	18.28 ± 0.87 a	18.86 ± 0.50 a	18.96 ± 0.65 a	20.1 ± 2.73 a	14.96 ± 8.36 c	14.22 ± 8.04 c	0.04
MCHC (%)	31.58 ± 0.55 a	31.66 ± 0.36 a	31.08 ± 0.62 a	31.08 ± 0.56 a	31.32 ± 0.22 a	32.18 ± 2.58 a	24.98 ± 13.97 b	24.08 ± 13.48 b	0.01
PLTs (10^3^/μl)	772.6 ± 51.56 b	857.8 ± 99.41 b	982.2 ± 225.22 a	905.2 ± 143.08 a	822.2 ± 83.55 a	1007.4 ± 242.84 a	779.8 ± 460.38 b	799.4 ± 486.61 b	0.01
WBCs (10^3^/μl)	14.43 ± 2.16 b	14.63 ± 3.98 b	14.38 ± 5.08 b	14.52 ± 5.64b	10.35 ± 3.89 c	18.12 ± 5.50 a	11.32 ± 6.70 c	17.44 ± 11.27 a	0.001
Neutrophils (10^3^/μl)	2.00 ± 0.13 c	1.73 ± 0.09 d	3.53 ± 15.49 b	4.00 ± 0.02b	2.9 ± 0.57 c	4.59 ± 0.78 b	5. 0 ± 0.77 a	9.55 ± 0.90 a	0.02
Lymphocytes (10^3^/μl)	10.63± 0.54 b	12.23± 0.32 a	9.97 ± 0.44 b	10.18 ± 0.91b	6..86 ± 0.74c	12.78 ± 1.62 a	4.86 ± 0.36 d	4.44 ± 0.77 d	0.001
Monocytes (10^3^/μl)	0.76 ± 0.05 b	0.41 ± 0.08 b	0.28 ± 0.04 c	0.17 ± 0.01 d	0.308 ± 0.07 c	0.36 ± 0.03 c	1.29 ± 0.09 a	3.13 ± 0.27 a	0.001
Eosinophils (10^3^/μl)	0.43 ± 0.03 a	0.26 ± 0.01 b	0.23 ± 0.03 b	0.05 ± 0.09 d	0.145 ± 0.09 c	0.05 ± 0.08 b	0.07 ± 0.01 d	0.069 ± 0.08 d	0.01
Basophiles (10^3^/μl)	0.61 ± 0.03 a	0.016 ± 0.01 d	0.38 ± 0.09 b	0.10 ± 0.06 c	0.135 ± 1.24 c	0.34 ± 0.07 b	0.144 ± 0.02 c	0.25 ± 0.02 b	0.01

Mean values ± SD having different lowercase letters within the same row are significantly different at *P* ≤ 0.05

The leukogram data demonstrated a stress picture of leukogram by a considerable (*P* ≤ 0.05) rise in WBCs in all AgNP-treatment groups after 15 days, while WBCs were considerably (*P* ≤ 0.05) increased after 30 days only in rat groups receiving 0.5 and 1 mg of AgNPs compared to the control group. Rats given 0.25 mg AgNPs after 30 days, on the other hand, exhibited no significant difference when compared to the control group. In terms of differential leukocyte count, the number of neutrophils increased significantly (*P* ≤0.05) in all AgNP-treated groups after 15 and 30 days as compared to the control group. The highest values were recorded in rats that received 1 mg AgNPs after 30 days. On the contrary, the number of lymphocytes decreased considerably (*P* ≤ 0.05) in all AgNP-treated groups after 15 and 30 days, with the exception of the rat that got 0.5 mg AgNPs after 30 days and had an enhanced lymphocyte count compared to the control group. The lowest values were recorded in rats that received 1 mg AgNPs after 30 days. Regarding monocyte number, there was a significant increase (*P* ≤ 0.05) in rats that got 1 mg AgNPs after 15 and 30 days but a significant decrease (*P* ≤ 0.05) in rats that received 0.25 and 0.5 mg AgNPs after 15 and 30 days of treatment as compared to the control group. The greatest value was obtained after 30 days of treatment in rats given 1 mg AgNPs. After 15 and 30 days of treatment, the number of eosinophils decreased significantly (*P* ≤ 0.05) in all treated groups compared to the control group. The lowest values were reported in rats that received 1 mg AgNPs after 30 days of exposure. Lastly, after 15 days, the number of basophils decreased significantly (*P* ≤ 0.05) in all treatment groups, although the drop was more pronounced in rats given 0.5 and 1 mg AgNPs. However, after 30 days of treatment, there was a substantial increase (*P* ≤ 0.05) in all of the rats given AgNPs when compared to the control rats. After 30 days of experimentation, the greatest results were obtained in the rat group that received 1 mg AgNPs. Table 2 shows the results of the leucogram.

### Serum biochemical findings

**Fig. 3:**
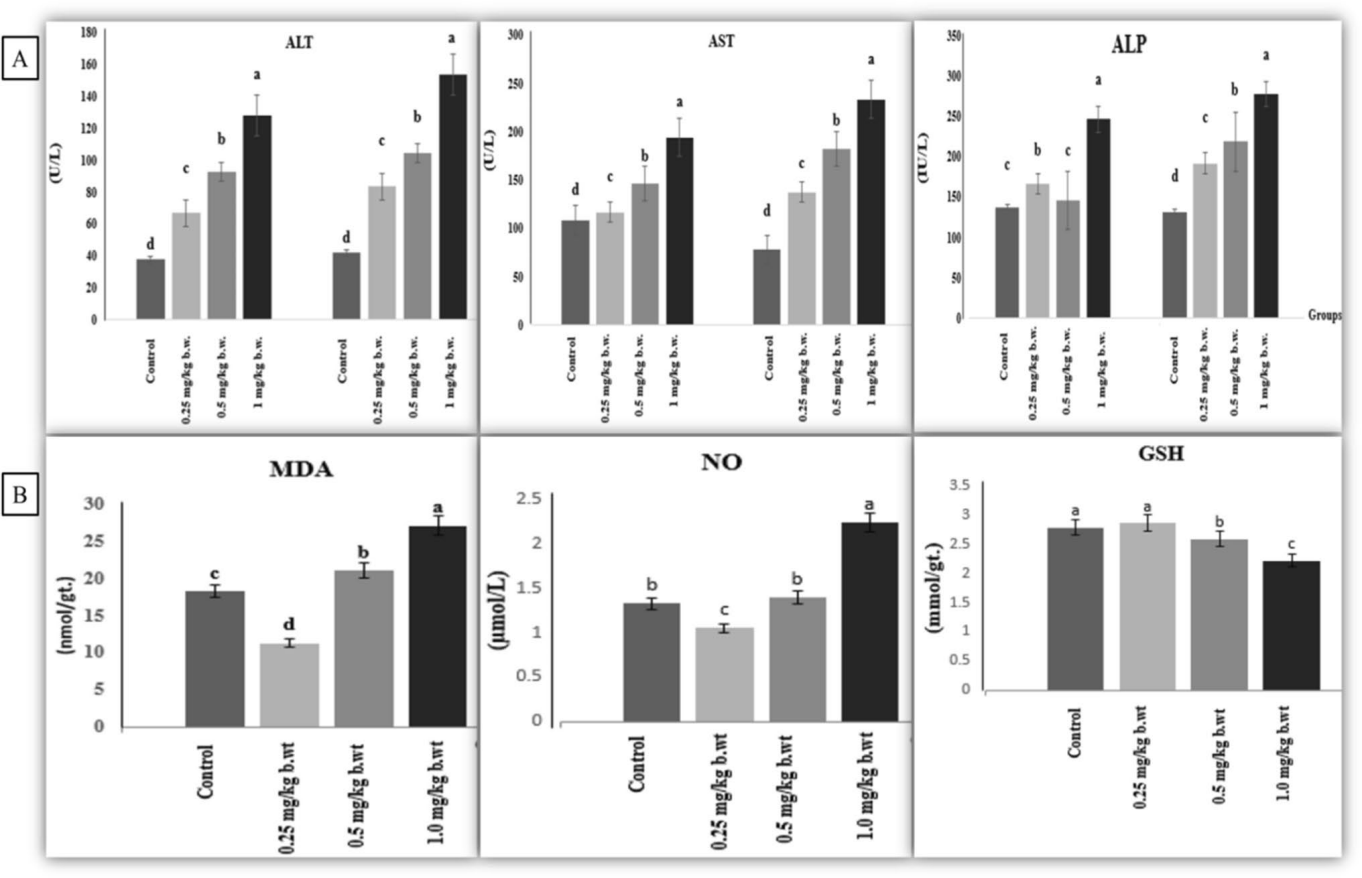
**a** Serum hepatic injury biomarkers alanine aminotransferase: ALT; aspartate aminotransferase (AST) and alkaline phosphatase: ALP of different groups of the experiment after 15 and after 30 days. **b** Hepatic oxidative and antioxidant biomarkers. Malondialdehyde: MDA; nitric oxide: NO and reduced glutathione: GSH. Values with different letters differ significantly at (*P* ≤ 0.05) *n* = 7

Figure 3 a shows the impact of AgNP treatment on serum hepatic injury biomarkers. After 15 and 30 days of treatment, the rats administered AgNPs showed a substantial (*P* ≤ 0.05) increase in serum enzymatic activity of ALT, AST and ALP compared to the control rats. The greatest rise was seen in rats given 1 mg of AgNPs after 30 days of treatment.

### Oxidative stress and antioxidant biomarkers

As seen in Fig. 3b, we detected a significant increase (*P* ≤ 0.05) in the hepatic level of MDA in rats given 0.5 and 1 mg AgNPs after 30 days of exposure as compared to the control group. Moreover, after day 30 of experiment, rats that received 0.25 mg AgNPs had a significant (*P* ≤ 0.05) decline in hepatic NO levels. However, the rats given 0.5 mg AgNPs showed no statistical changes compared to the control group. However, the rat group that received 1 mg AgNPs exhibited a significant increase (*P* ≤ 0.01) in NO levels after 30 days of exposure as compared to the control group. In contrast, following 30 days of exposure to AgNPs, the hepatic GSH content significantly decreased (*P* ≤ 0.05) in rats injected with 0.5 and 1 mg compared with the control group, while the lowest value was recorded in rats that received 1 mg AgNPs.

### Histopathological findings

**Fig. 4:**
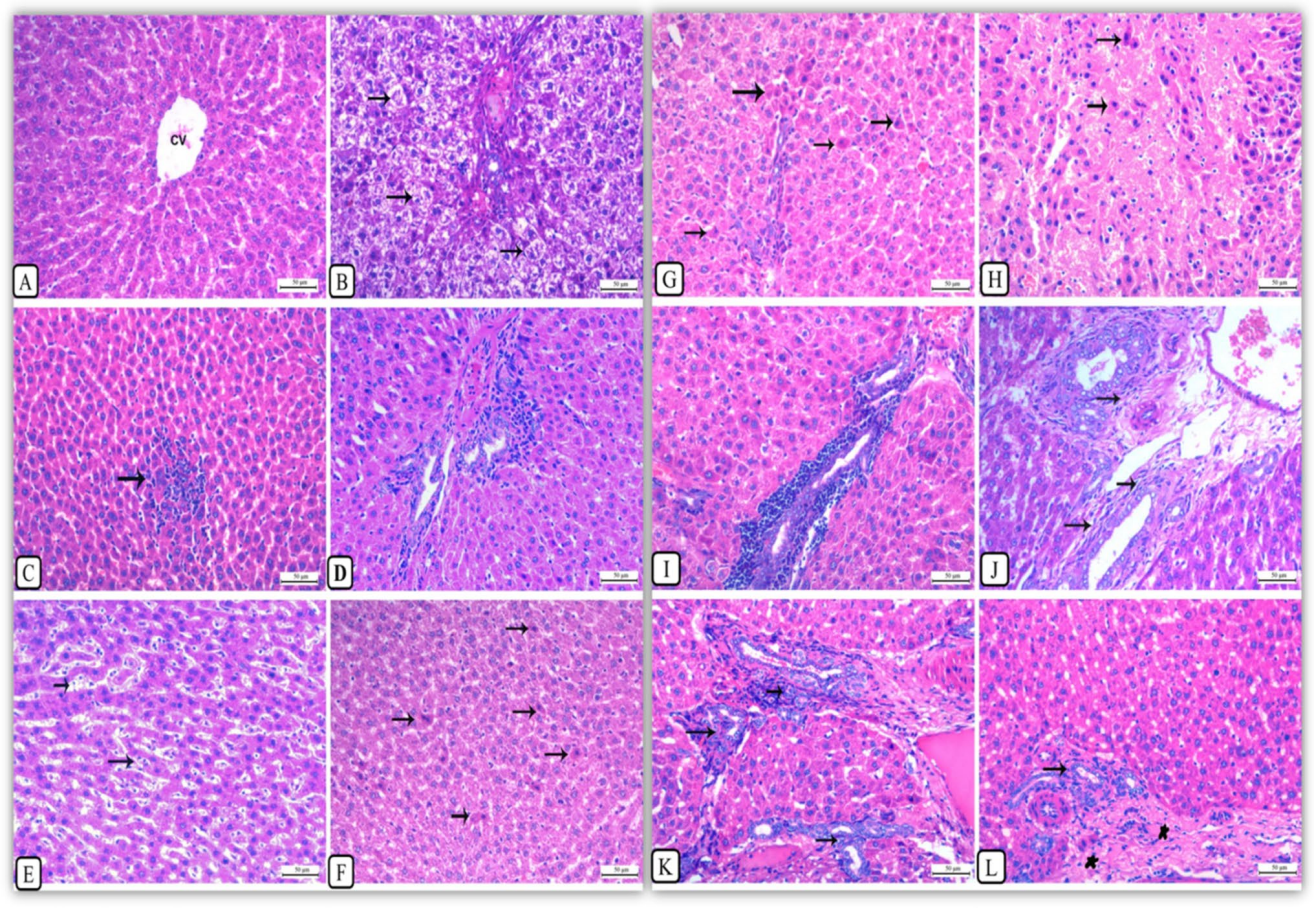
Liver, first sacrifice: after 15 days exposure of AgNPs. **a** Control rats showed normal hepatic parenchyma of the central vein (CV) with radiating hepatic cords. **b** Rats received 0.5 mg/kg b.w. AgNPs, showing marked cytoplasmic vacuolation of hepatocytes with pyknotic nucleus (arrows). **c** Rats received 0.5 mg/kg b.w. AgNPs, showing the focal area of hepatic necrosis accompanied by mononuclear cell infiltrations and sinusoidal cell activation (arrow). **d** Rats received 0.5 mg/kg b.w. AgNPs, showing an increased degree of portal bile ducts’ hyperplasia with periductal and perivascular cellular reaction. **e** Rats received 1 mg/kg b.w. AgNPs, showing marked activation of sinusoidal cells (arrows). Liver, first sacrifice: after 30 days of exposure of AgNPs. **f** Rats received 1 mg/kg b.w. AgNPs, showing apoptotic changes in some hepatocytes with deep eosinophilic cytoplasm and pyknotic nucleus (arrows). Liver, second sacrifice after 30 days of exposure of AgNPs. **g** Rats received 0.25 mg/kg b.w. AgNPs: showing marked individual hepatic cell apoptosis with condensed cytoplasm and pyknotic nucleus (arrows). **h** Rats received 0.5 mg/kg b.w. AgNPs: showing marked diffuse hepatic coagulative necrosis with the surrounding hepatocytes showing apoptosis (arrows). **i** Rats received 0.5 mg/kg AgNPs showing marked peri-vascular and periductal mononuclear cell infiltration, most probably lymphocytes. **j** Rats received 0.5 mg/kg AgNPs showing marked proliferation of fibroblasts around the hyperplastic bile ducts (arrows). **k** Rats received 1 mg/kg AgNPs: showing marked proliferation of bile ducts. **l** Rats received 1 mg/kg AgNPs showing massive periductal (arrow) and perivascular collagen deposition, stars. All are H&E stained, bar = 50μ (×200)

The liver tissues of untreated control rats showed normal hepatic parenchyma of the central vein with radiating hepatic cords (Fig. 4a) and normal portal area.

#### Histopathological findings after 15 days of exposure to AgNPs

On the other hand, rats treated with AgNPs showed dose- and time-dependent damages in their livers which were manifested as inflammatory, degenerative, necrotic and hyperplastic changes. The observed hepatic alterations began as mild in the form of hyperemia of either the central vein or sinusoids, some focal areas of haemorrhages, cytoplasmic vacuolar degeneration of hepatocytes (Fig. 4b), small early focal areas of hepatic necrosis accompanied by mononuclear cells infiltrations (Fig. 4c) and perivascular and periductal infiltrations with mild bile ducts hyperplasia (Fig. 4d), along with proliferation of sinusoidal (Fig. 4e) and Ito cells with thickening of the hepatic capsule accompanied by mononuclear cell infiltration but not invading the hepatic parenchyma; subcapsular steatosis were also seen as compared with control rats. In addition, some hepatocytes showed apoptotic changes in the form of deeply eosinophilic cytoplasm with pyknotic darkly stained nuclei (Fig. 4f).

#### Histopathological findings after 30 days of exposure to AgNPs

Hepatic lesions were more pronounced and varied from individual hepatocellular necrosis and apoptosis where the cytoplasm of hepatocytes appeared deeply eosinophilic with pyknotic nuclei (Fig. 4g) to multiple focal necrotic areas accompanied by a notable mononuclear cell infiltration or widespread hepatic coagulative necrosis (Fig. 4h). Obvious biliary hyperplasia was noted extending into the hepatic parenchyma with newly formed small bile ductules associated with periductal mononuclear cell infiltration (Fig. 4i) and onset of fibroplasia (Fig. 4j). By dose grading, a prominent biliary hyperplasia (Fig. 4k) with increasing periductal fibroplasia and collagen deposition was observed (Fig. 4l). The degree and severity of these alterations were dose dependent as shown in Table 3.

**Table 3:** Hepatic histopathological lesions of the first sacrifice of rats after 15 and 30 days of i.p. injection of AgNPs

	Group B (0.25 mg/kg b.w.)	Group C (0.5 mg/kg b.w.)	Group D (1 mg/kg b.w.)
Hepatic lesions after 15 days from AgNP exposure			
Congestion	**+/++**	+/++	+++
Capsular thickening	**++**	++	++
Vacuolar degeneration	+	+++/ ++++	+
Sinusoidal, Ito cell activation	++	+++	+++
Individual cell necrosis (apoptosis)	+	++	++
Focal necrosis	+	+	+++
Bile duct hyperplasia	+	++	+++
Periductal/perivascular reaction	+	++	+++
Hepatic atrophy	**-**	-	+
Silver pigment deposition	+	++	+
Hepatic lesions after 30 days from AgNP exposure			
Pericentral congestion/H. atrophy	**++**	-	-
Vacuolar degeneration	(fatty change) +	+	-
Hepatic double nuclei	+	+++	+++
Mitotic figures	-	+	++
Sinusoidal, Ito cell activation	++	+++	+++
Individual cell necrosis	+	++	++++
Focal necrosis	++	+++	+++
Bile duct hyperplasia	++++	++++	++++
Biliary fibroplasia	-	-	++
Periductal/perivascular reaction	++++	++++	++++
Vascular degeneration	+	++	Wall +++ hyalinization
Nano silver pigment	+	+	+

-, negative; +, mild; ++, moderate; +++/++++, severe

#### Masson’s trichrome staining

**Fig. 5:**
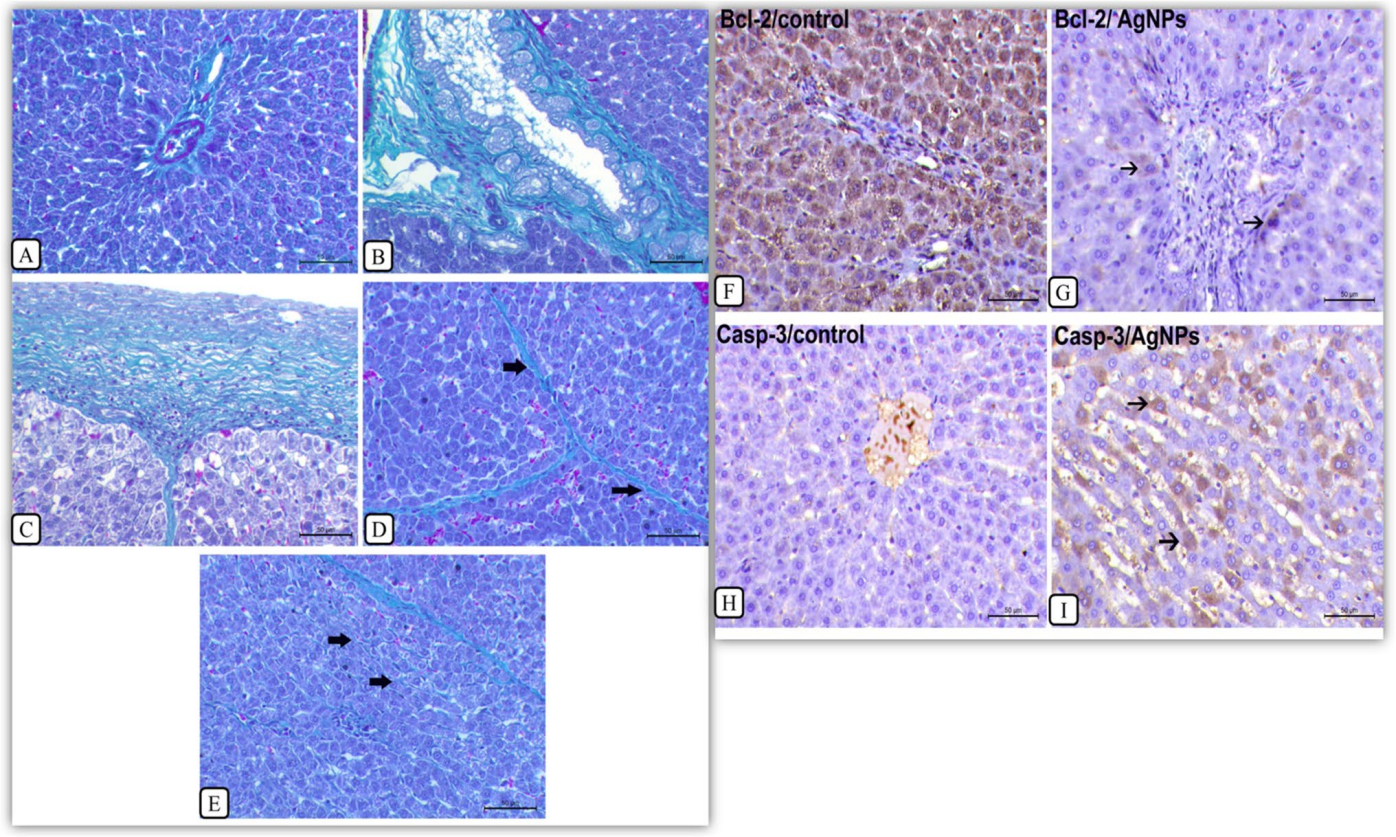
Masson’s trichrome staining for the liver, detection of collagen deposition, and rats receiving 1 mg/kg AgNPs for 30 days. **a** Control rats, liver showing a small amount of fibrous tissue in the portal area. **b** Rats treated with AgNPs, liver showing a marked increase in fibrous tissue deposition in the portal area which shows a remarkable bile duct hyperplasia. **c** Rats treated with AgNPs, liver showing a marked thickening of the hepatic capsule with extension of fibrous tissue into the hepatic parenchyma (arrow). **d** Rats treated with AgNPs, liver showing interlobular proliferation of fibrous connective tissue (arrows). **e** Rats treated with AgNPs, liver showing intrahepatic proliferation of fibrous connective tissue (arrow), bar = 50 μ (×200). **f–i** Immunolabelling of Bcl-2 and caspase-3 in the liver tissues of rats treated with AgNPs for 30 days. **f** Control liver, showing a strong cytoplasmic expression of Bcl-2. **g** Rats treated with AgNPs, liver showing a negative expression of Bcl-2 except of faint cytoplasmic labelling in few cells (arrows). **h** Control liver, showing a negative cytoplasmic expression of Caspase-3. **i** Rats treated with AgNPs, liver showing strong cytoplasmic labelling of caspase-3. Immunohistochemistry, bar = 50 μ (×200)

The control untreated rats showed a small amount of fibrous tissue in the portal area (Fig. 5a). However, the rats injected with AgNPs for 30 days showed a marked increase in fibrous tissue deposition in the portal area around the proliferating bile ducts (Fig. 5b). Also, a marked thickening of the hepatic capsule with extension of fibrous tissue into hepatic parenchyma was noticed (Fig. 5c) accompanied by proliferation of fibrous connective tissue either interlobular (Fig. 5d) or intralobular (Fig. 5e).

### Immunohistochemical labelling of Bcl-2 and Caspase-3 in the liver tissues

Immunolabelling of Bcl-2 and Caspase-3 in the liver tissues of rats treated with AgNPs for 30 days at a dose of 1 mg/kg b.w revealed that the control group liver exhibited strong cytoplasmic immunoreactivity (*P* < 0.05) of Bcl-2 (Fig. 5f) while the liver of rats treated with AgNPs showed a negative expression of Bcl-2 except of faint cytoplasmic labelling in individual hepatocytes (Fig. 5g). The control rat liver revealed a negative cytoplasmic expression of caspase-3 (Fig. 5h) while the liver of rats treated with AgNPs demonstrated a moderate positive cytoplasmic labelling (*P* < 0.05) of caspase-3 (Fig. 5i).

### Gene expression analysis

**Fig. 6:**
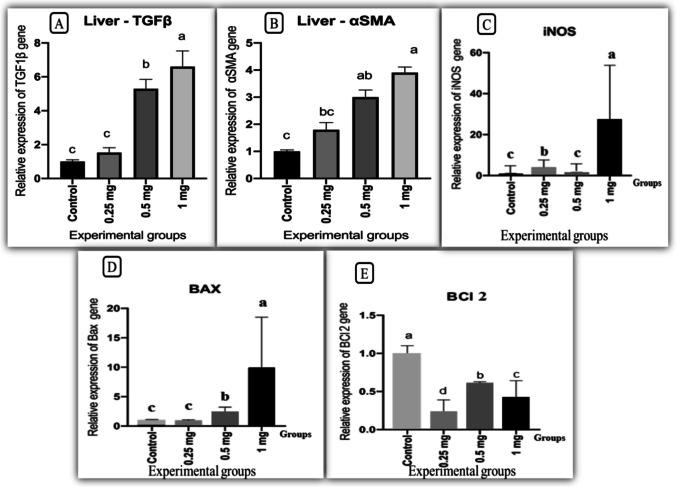
Effect of AgNPs on expression analysis of transforming growth factor: TGFβ1, induced nitric oxide synthase: iNOS, alpha smooth muscle: αSMA, BAX, and Bcl-2 genes in livers of rats after 30 days of exposure (*n* = 8). Bars with a, b, c letters are significantly different from each other (*P* < 0.05)

Our findings demonstrated that AgNP administration altered the expression analysis of TGF-β1 and αSMA, iNOS, Bax and Bcl-2 genes. TGF β1and αSMA expression levels as markers for hepatic fibrosis markers were significantly upregulated (*P* ≤ 0.01) in rat groups given 0.5 and 1 mg AgNPs while they were non-statistically significant in rats given 0.25 mg AgNPs as compared to the control group (Fig. 6a, b). iNOS expression levels as inflammatory and nitrosative stress markers were increased significantly (*P* ≤ 0.01) in rat groups given 0.25 and 1 mg AgNPs while they were non-statistically significant in rats receiving 0. 5 mg AgNPs compared to the control group (Fig. 6c). Furthermore, poptotic/apoptotic markers and Bax expression levels were significantly elevated (*P* ≤ 0.01) in rat groups given 0.5 and 1 mg AgNPs while they were statistically non-significant in rats given 0.25 mg AgNPs as compared to the control group (Fig. 6d). On the other hand, B-cell lymphoma 2 expression levels were significantly (*P* ≤ 0.05) downregulated in all AgNP-treated groups as compared to the control group (Fig. 6e).

## Discussion

The goal of this study is to investigate the effects of intraperitoneal injections of AgNPs in various doses and durations on all body physiological processes, as well as their impact on histological architecture and body defence mechanisms. In previous toxicological studies, the size of AgNPs is important (Li et al. 2020; Hassanen et al. 2019; Chen et al. 2015; Park et al. 2011). AgNPs measuring 10 nm in diameter were toxic, whereas 40–75-nm AgNPs were not (Gliga et al. 2014). The current investigation discovered that a reduction in body weight was the first symptom of AgNP toxicity, especially in rats administered 0.5 and 1 mg compared to the control group. as stated by De Jong et al. (2013) and Zhang et al. (2013). However, Luaibi and Qassim (2020) observed a significant elevation in total body weights of female SD rats chronically exposed to AgNPs. The varying effects on b.w. may be influenced by the method of exposure, which differs between I/V injection, oral gavage and inhalation (Lee et al. 2018). After 15 and 30 days of exposure to the maximum dose of AgNPs, we discovered a drop in liver weight which might be attributed to a striking rise in lipid peroxidation, leading to structural changes to lipid vacuoles (Rezaei et al. 2018). The haematological profile can be an effective tool for monitoring health status (Imani et al. 2014). In this investigation, AgNPs lowered the RBC count, Hb and HCT in all treated groups in a dose-dependent way. However, MCV, MCH and MCHC declined and indicated a microcytic hypochromic anaemia only in the high dose–treated group compared with the control group. Our results were reinforced by the findings of De Jong et al. (2013), Lee et al. (2018), Rezaei et al. (2018), Forouhar Vajargah et al. (2019) and Al-Baker et al. (2020). The noted variations in erythrogram findings may be attributed to the increased immunogenic response or the disrupted signalling pathways and cell maturation (Sarhan and Hussein 2014), or because of the negative impact on the hematopoietic organs (Atmaca et al., 2014). White blood cells play an essential role in the immune response to infection as well as allergic or toxic reactions to drugs or chemicals (Park et al. 2010). The current study found that 15 and 30 days of AgNP exposure resulted in a marked increase in WBCs in all treated groups compared with the control group, particularly in rats treated with 0.5 and 1 mg AgNPs. Such observations were consistent with Tiwari et al. (2011) and Qin et al. (2017). The increase in WBCs following AgNP exposure is considered a typical reaction in rats due immune system activation for AgNP phagocytosis (Li et al. 2017; Hassanen et al. 2019) or may be due to stress responses caused by AgNP injection (Hadrup and Lam 2014); thus, AgNPs can sharply alter the lymphocyte/granulocyte ratios (Aniya et al. 2005; Tang et al. 2009). In this work, rats injected with 0.25 mg and 0.5 mg AgNPs had a substantial increase in PLT count in a dosage- and time-dependent way; however, rats injected with 1 mg AgNPs showed no significant changes in PLT count when compared to the control group. Kim et al. (2010) demonstrated that after 30 weeks, oral treatment of AgNPs had no effect on the prothrombin time (PT) and activated partial thromboplastin time (APTT) of rat plasma. Furthermore, it was shown that exposure of human plasma to a level up to 40 μg/ml AgNPs did not cause a significant alteration in the coagulation process (Huang et al. 2016). On the other hand, several researches have shown that AgNPs boost coagulation activity via the intrinsic pathway (Martínez-Gutierrez et al. 2012) aggregation of platelets, thrombin production and increased expression of P-selectin, phosphatidylserine and intracellular calcium (Jun et al. 2011; Laloy et al. 2014). The liver is a target site of NP deposition (Kim et al. 2008). Many investigations have found that AgNPs cause cytotoxicity, DNA damage and apoptosis mediated by enhanced lipid peroxidation and development of oxidative stress (AshaRani et al. 2009; Xin et al. 2014; Zhu et al. 2017; Sooklert et al. 2019). We further demonstrate that oxidative stress may be implicated in liver damage produced by AgNP injections that facilitated the liberation of serum enzymes AST, ALT and ALP as biomarkers for liver damage (Abdelhady et al. 2017; Assar et al. 2021; Abd El Latif et al. 2021). These observations were side by side with the findings of Qin et al. (2017), Lee et al. (2018), Abd El-Maksoud et al. (2019) and Odeyemi et al. (2019). AgNPs caused toxicity through promoting lipid peroxidation (Ahmed and Hussein 2017; Rezaei et al. 2018; Li et al. 2020). Increased ROS levels can disrupt the intracellular redox balance resulting in oxidative stress, inflammation and ultimately cell death (Xia et al. 2006). Our findings showed that a state of oxidative injury was provoked by AgNPs in a dose-dependent way as evidenced by the raised hepatic MDA levels with the depletion of antioxidant defensive mechanism by reducing the hepatic GSH levels. The most severe hepatic damage was associated with increasing AgNP-given dose and expanding exposure time. Piao et al. (2011), Xin et al. (2014), Zhu et al. (2017), Li et al. (2017), Srivastava et al. (2012), Ansar et al. (2017) and Li et al. (2020) have all stated that continuous elevation of Ag^+^ concentration leads to continuous induction of hydroxyl radical, which consumes much more intracellular GSH, disturbing the homeostasis of free radical scavenging (Matés 2000; Srivastava et al. 2012). Similar investigations found that AgNPs reduced CAT activities and raised MDA levels in fish liver, causing oxidative damage (Wu and Zhou 2013), and in rats (Adeyemi and Adewumi 2014). Fatemi et al. (2017) found a considerable inhibition in GSH level and GPX activity with a marked elevation in MDA level by AgNP exposure. Many studies approved that the liver is the main target organ for AgNP action (Ji et al. 2007; Gopinath et al. 2010; Kim et al. 2008 and Sung et al. 2009). The histological assessment of the liver indicated pathological alterations which were dose and time dependent that grew more severe in the liver after 30 days of exposure and were graded with increasing concentrations of AgNPs. Bile-duct hyperplasia represents the entire mark of the histological alterations of AgNPs on the liver, which was compatible with the pathologic data in the 28-day oral toxicity (Kim et al. 2008) and 90-day inhalation (Sung et al. 2009) and inflammation in liver tissue in accordance with Lee et al. (2013a, b) who found a mild infiltration of inflammatory cells in the portal vein area owing to the AgNP build-up in macrophages (Kupffer cells). In this study, after 30 days of exposure to 1 mg AgNPs, various hepatocellular alterations such as coagulative hepatic necrosis associated with mononuclear cell infiltration and marked apoptosis were noted as documented by Albrahim and Alonazi (2020). The most consistent and identifiable pathological lesions identified in all AgNP-treated groups were capsular thickening, sinusoidal thickening and Ito cell proliferation. The present study’s findings support the previous study’s conclusions that AgNPs cause liver damage (Sung et al. 2009; Kim et al. 2010). Fatemi et al. (2017) also detected widely dilated blood vessels, congested hepatic sinusoids filled with RBCs and a vacuolated appearance of liver tissues where sinusoidal cells are important for removing NPs (Sadauskas et al. 2007). The current study found that sinusoidal cell activation was dosage dependent, which is corroborated by Korani et al. (2011). As a result, the quantity of Kupffer cells in liver tissue can represent the degree of damage. Furthermore, Loghman et al. (2012) found that AgNPs generated mitochondrial cytotoxicity by raising the AgNP concentration, significantly lowering mitochondrial function, increasing membrane leakage and inducing necrosis and apoptosis (Hussain et al. 2005; Yang et al. 2017). Inflammatory mononuclear cell infiltration was associated with AgNP toxicity, which may be caused by increased cytokine production (Wen et al. 2017). Apoptosis is known as programmed cell death, which is a gene-regulated event where hepatocyte apoptosis can result in malfunction, suppression of proliferation, cycle arrest and diminished viability, resulting in liver fibrosis (Zhou et al. 2014). Apoptosis is regulated by numerous methods, including death receptors, caspase activation, mitochondrial responses and the control of BAX gene expression (Ahmadian et al. 2018a; b). AgNP-mediated apoptosis was linked to the intrinsic mitochondrial-dependent mechanism. Furthermore, the induction of apoptotic cell death may be assessed using marker genes such as B-cell lymphoma 2 (Bcl-2) which is an apoptotic inhibitory factor, whereas the BAX gene was identified as an apoptotic gene that promotes apoptosis by heterodimerization or homodimerization with Bcl-2 (Zeng et al. 2014; Jiang et al. 2018). Caspase-3 is a well-known apoptotic marker that may be triggered by both extrinsic and intrinsic apoptotic pathways, resulting in DNA breakage (Lee et al. 2014). Nanoparticles are thought to disrupt the cellular immune system, increasing the vulnerability to apoptosis (Halappanavar et al. 2011; Zhang et al. 2012; Park and Yeo 2013; Naeemi et al. 2020). Currently, immunohistochemical analysis showed a significant decrease in Bcl-2 immunolabelling with significant upregulation of caspase-3 in the hepatic tissues of AgNP-treated rats as compared with the control group. Additionally, a significant elevation in the mRNA expression level of the BAX gene in rats given 0.25 and 0.5 mg AgNPs compared with the control group was observed, with the greatest expression associated with the highest administered dose of AgNPs; however, all AgNP-treated groups showed a significant downregulation of the Bcl-2 gene. These findings are backed up by Piao et al. (2010), Piao et al. (2011), Jeyaraj et al. (2015), Bastos et al. (2017) and Chauhan et al. (2019). These findings align with those recorded by Zielinska et al. (2018) who discovered that AgNPs suppressed Bcl-2 expression in PANC-1 cells considerably. Furthermore, Hassanen et al. (2019) found caspase-3 immunoreactivity in the hepatic tissues of rats treated with AgNPs for 14 days and 28 days (Pourhamzeh et al. 2016). Recent research found caspase-3 overexpression in the hepatic and renal tissues of rats subjected to 50 mg AgNPs/kg b.w (Shehata et al. 2021). The expression of caspase-3 is a dependable marker of apoptosis in the rat liver (Eckle et al. 2004) and in HepG2 cells (Xue et al. 2018). One of the most intriguing findings of the current study was the higher levels of the iNOS gene after 30 days of exposure to 1 mg AgNPs. This is because iNOS is one of three nitric oxide synthase (NOS) enzymes responsible for nitric oxide generation, which plays a crucial role in a variety of biological activities (Xue et al. 2018). Nitric oxide generation is linked to tissue fibrosis because it induced fibrogenic cytokines, increased collagen synthesis (Prandota 2010) and enhanced NO production in rats given 1 mg AgNPs whereas their livers revealed substantial fibrosis and collagen deposition, with a significant upregulation of the iNOS gene in all treatment groups when compared to the control. This observation is consistent with Weber et al. (2003) and Badr et al. (2019). Transforming growth factor beta 1 gene is considered a potent pro-fibrogenic factor (Arslan et al. 2010; Polimeni et al. 2016). Alpha smooth muscle actin gene (α-SMA) is a marker of myofibroblasts and has an important role in fibrosis (Tomasek et al. 2002) and is used as a marker for the fibrogenic activity of activated tissue (Bochaton-Piallat et al. 2016). However, prolonged or excessive myofibroblast activity may result in fibrosis and organ dysfunction (Kong et al. 2014; Travers et al. 2016). So, the expression of α-SMA correlates with the activation of myofibroblasts. In the existing study, a significant upregulation of TGF-1β and α-SMA genes was noticed in all treated groups in a dose-dependent manner as compared with the control group. Our result agrees with that of Tian et al. (2007), Wang et al. (2014), Martin et al. (2019) and Zhou et al. (2021). Jain et al. (2013) showed a positive correlation between TGF-β expression and the increased ROS production after AgNP treatment and triggering hepatic apoptosis (Sancho et al. 2012) and fibrosis together with enhanced expression of α-SMA (Sterreicher et al. 2011).

## Conclusion

According to the current findings, intraperitoneal injection of AgNPs in various upgrading concentrations for 15 and 30 days induced hepatic histopathological changes associated with disturbance in haematological and biochemical parameters as well as disruption of gene expression analysis. Our findings give a wider and more precise prospective knowledge of the molecular processes underpinning AgNP-induced chronic hepatic inflammation and fibrosis. As AgNP ip treatment increased hepatic MDA and NO while depleting hepatic GSH content, it also triggered the endogenous apoptotic pathway by upregulating the expression of Bax and caspase-3 while decreasing the antiapoptotic factor Bcl2, in addition to the inclusion of iNOS which increased the hepatic inflammatory process by inducing hepatic fibrosis via the TGFβ-1 and αSMA pathway.

## Data Availability

The authors confirm that the data supporting the findings of this study are available within the article [and/or] its supplementary materials.
